# Advances of exosomes in diabetic wound healing

**DOI:** 10.1093/burnst/tkae078

**Published:** 2025-02-20

**Authors:** Weixue Jin, Yi Li, Meirong Yu, Danyang Ren, Chunmao Han, Songxue Guo

**Affiliations:** Department of Plastic Surgery, Second Affiliated Hospital of Zhejiang University School of Medicine, 1511 Jiang Hong Road, Binjiang District, Hangzhou 310009, Zhejiang, China; Department of Plastic Surgery, Second Affiliated Hospital of Zhejiang University School of Medicine, 1511 Jiang Hong Road, Binjiang District, Hangzhou 310009, Zhejiang, China; Center for Basic and Translational Research, Second Affiliated Hospital Zhejiang University School of Medicine, 88 Jie Fang Road, Shangcheng District, Hangzhou 310009, Zhejiang, China; Department of Plastic Surgery, Second Affiliated Hospital of Zhejiang University School of Medicine, 1511 Jiang Hong Road, Binjiang District, Hangzhou 310009, Zhejiang, China; Department of Burns and Wound Repair, Second Affiliated Hospital Zhejiang University School of Medicine, 88 Jie Fang Road, Shangcheng District, Hangzhou 310009, Zhejiang, China; Zhejiang Key Laboratory of Trauma, Burn, and Medical Rescue, 88 Jie Fang Road, Shangcheng District, Hangzhou 310009, Zhejiang, China; Department of Plastic Surgery, Second Affiliated Hospital of Zhejiang University School of Medicine, 1511 Jiang Hong Road, Binjiang District, Hangzhou 310009, Zhejiang, China; Zhejiang Key Laboratory of Trauma, Burn, and Medical Rescue, 88 Jie Fang Road, Shangcheng District, Hangzhou 310009, Zhejiang, China

**Keywords:** Wound healing, Diabetes, Exosome, Extracellular vesicle, Biomaterials

## Abstract

Poor wound healing is a refractory process that places an enormous medical and financial burden on diabetic patients. Exosomes have recently been recognized as crucial players in the healing of diabetic lesions. They have excellent stability, homing effects, biocompatibility, and reduced immunogenicity as novel cell-free therapies. In addition to transporting cargos to target cells to enhance intercellular communication, exosomes are beneficial in nearly every phase of diabetic wound healing. They participate in modulating the inflammatory response, accelerating proliferation and reepithelization, increasing angiogenesis, and regulating extracellular matrix remodeling. Accumulating evidence indicates that hydrogels or dressings in conjunction with exosomes can prolong the duration of exosome residency in diabetic wounds. This review provides an overview of the mechanisms, delivery, clinical application, engineering, and existing challenges of the use of exosomes in diabetic wound repair. We also propose future directions for biomaterials incorporating exosomes: 2D or 3D scaffolds, biomaterials loaded with wound healing-promoting gases, intelligent biomaterials, and the prospect of systematic application of exosomes. These findings may might shed light on future treatments and enlighten some studies to improve quality of life among diabetes patients.

Highlights

- This review provides an overview of the mechanisms, delivery, clinical application, engineering, and existing challenges of exosomes in diabetic wound repair.
- We propose future directions for biomaterials incorporating exosomes: 2D or 3D scaffolds, biomaterials loaded with wound healing-promoting gases, intelligent biomaterials (e.g. pH-responsive, photothermal-responsive, etc.), and the prospect of systematic application of exosomes, which may enlighten future research to develop engineered biomaterials such as scaffolds or microneedles for sustained release of exosomes.
- We recapitulate the clinical research advancements regarding exosomes in wound healing, and propose the dilemma of clinical application and corresponding solutions.

## Background

A typical reaction to injury is wound healing, which involves a variety of cellular and molecular players in a dynamic process [1]. One of the most intractable issues for doctors is poor diabetic wound healing, which also places significant financial and physical strain on patients. Diabetes affects 285 million people (aged 20–79 years), with projected growth to 439 million people by 2030 [1]. It is estimated that diabetic foot ulcers (DFUs) cause ~50%–70% of all limb amputations [2]. Approximately 30% of people with diabetic foot ulcers die within 5 years, and the rate increases to almost 70% in cases of serious amputation [3]. Diabetes mellitus (DM) can hinder the healing of wounds and may trigger chronic nonhealing foot ulcers, which can result in mortality and severe depression [2]. Promotion of wound healing is a viable tactic for reducing amputations and fatalities among diabetes patients. Growth factor therapy, cytokine stimulators or inhibitors, gene therapy, and other conventional wound healing procedures do not produce adequate outcomes [4]. Furthermore, the likelihood of pigmentary abnormalities and atrophic scarring is increased by traditional procedures such as biological stenting, skin grafting, skin flap transplantation, and laser therapy [4]. Therefore, the demand for more effective and secure wound-healing techniques is critical.

**Figure 1:**
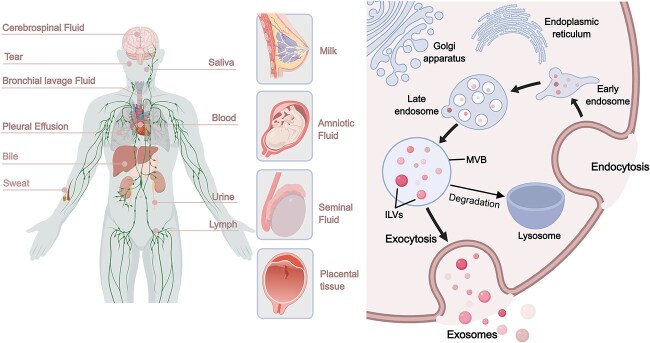
Biogenesis and sources of exosomes. Exos are found in practically every physiological fluid, including urine, blood, sweat, saliva, tears, bile, and lymph, cerebrospinal fluid, semen, breast milk, amniotic fluid, and placenta. Exosome biogenesis involves double invagination of the plasma membrane and the formation of MVBs containing ILVs. ILVs are eventually released as exosomes via the fusion of MVBs to the plasma membrane and exocytosis (Created with MedPeer). *ILVs* intraluminal vesicles, *MVB* multivesicular body

Exosomes (Exos), a group of extracellular vesicles (EVs), contain a variety of bioactive substances such as proteins, lipids, messenger RNAs, microRNAs (miRNAs), transfer RNAs, long noncoding RNAs, and mitochondrial DNA. Owing to their bioactive components, these bioactive materials can reprogram recipient cells [5–7]. Exosome biogenesis is a meticulously monitored procedure in which the plasma membrane is doubly invaginated and intracellular multivesicular bodies (MVBs) harboring intraluminal vesicles (ILVs) are formed. Exos, which range in diameter from 40 to 160 nm, are ultimately liberated from ILVs through the fusion of MVBs with the plasma membrane and exocytosis, releasing these nanosized biovesicles into bodily fluids [5,7,8]. The physicochemical properties of exosomes, particularly their size, shape, density, porosity, and surface charge, must be evaluated to determine their biological interactions [9,10]. It is becoming increasingly crucial to have the capacity to extract exosomes in an efficient and cost-effective manner for scientific study and clinical diagnostics. Currently, biophysical, molecular, and microfluidic techniques, such as dynamic light scattering, nanoparticle tracking analysis, transmission electron microscopy, atomic force microscopy, flow cytometry, tunable resistive pulse sensing, surface plasmon resonance (SPR)-based nanosensors, and nano-DLD (deterministic lateral displacement) are utilized to characterize exosomes [4,9,11,12]. Exos are found in practically every physiological fluid, including urine, blood, serum, saliva, bile, lymph, breast milk, cerebrospinal fluid, amniotic fluid, and malignant ascites, in both healthy and diseased patients [13] (Figure 1).

The majority of cell types, including immune cells (B cells, T cells, dendritic cells, and mast cells), endothelial cells (ECs), epithelial cells, neuronal cells, embryonic cells, cancer cells, and mesenchymal stem cells (MSCs), release exosomes [13]. *In vitro* bioinformatic analysis revealed that exosomes originating from bone marrow mesenchymal stem cells (BMSCs) primarily stimulate cell proliferation, whereas adipose mesenchymal stem cell (ADSC)-derived exosomes (ADSC-exos) have a more significant effect on angiogenesis [14]. The BMSC-EVs had the greatest amounts of fibroblast growth factor 2 (FGF-2) and platelet-derived growth factor BB and the most potent effects on fibroblasts. Umbilical cord MSCs contain the greatest amount of transforming growth factor beta (TGF-β) and have the greatest effect on keratinocytes [15]. According to previous studies, MSCs are frequently employed because of their regenerative capabilities and therapeutic potential [16–19]. However, stem cell treatment may cause storage and transportation issues, together with the hazards of cancer and deformity [20]. Exos have demonstrated therapeutic promise comparable to that of their parental cells and have application potential in the clinic. They communicate functional cargos (e.g. growth factors, cytokines, and miRNAs) from parental cells to target cells, altering the biological processes of the recipient skin cells [10]. They have shown paracrine therapeutic effects as a novel cell-free approach, enhancing regenerative phenotypic traits, managing inflammation, altering neovascularization and reepithelialization, and encouraging fibroblast activation and collagen formation, thereby increasing skin regeneration and wound repair [8,10,21]. One of the benefits of exosome treatment is a decreased risk of tumorigenesis and deformity compared to stem cell treatment. Exos are stable and simple to store [22]. Furthermore, they have homing effects and reduced immunogenicity, and their dose is readily regulated [4,7]. Owing to their advantageous biological properties and effective molecular cargo exchange, exosomes are highly suitable for skin tissue engineering and regenerative therapy in diabetes.

## Review

### Wound healing process in DM

For optimum wound healing, a well-orchestrated concordance of the overlapping biological and molecular activities of cell migration, reepithelialization, angiogenesis, and extracellular matrix (ECM) deposition and remodeling is necessary [23]. These processes entail a complicated interaction between cells and the ECM and can manifest as scarring or tissue regeneration [9,24]. Coagulation, the first stage of healing, kicks in right away to stop the bleeding after skin trauma. The major participants are platelets and the initiation of the coagulation cascade, with fibrin chains binding within the first few seconds [21]. Ultimately, thrombin converts platelet aggregates into a stable clot, which contributes significantly to the formation of a fibrin mesh [10]. During the inflammatory phase, growth factors, enzymes, chemokines, and antimicrobial agents secreted by leukocytes (neurophils, monocytes, T cells, macrophages and others) drawn into this spot cause swelling, heat, redness, and pain [21,25]. During the proliferative stage, reepithelialization occurs when epithelial migration continues until the wound is fully covered and an unbroken epithelial barrier is rebuilt [4]. Fibroblasts that have been stimulated and differentiated into myofibroblasts begin to compress the wound margins, the process of which is stimulated by M2 macrophages [26,27]. Smooth muscle actin (a-SMA) expression is characteristic of myofibroblasts [28]. Furthermore, fibroblasts start releasing large amounts of immature type III collagen into the matrix [29]. Finally, in the remodeling phase, type I collagen gradually replaces type III collagen, and the wounded location is covered by fresh tissues and scar tissues. Tissue remodeling subsequently results in progressive improvements in strength and flexibility via epithelialization and neovascularization [30].

**Figure 2:**
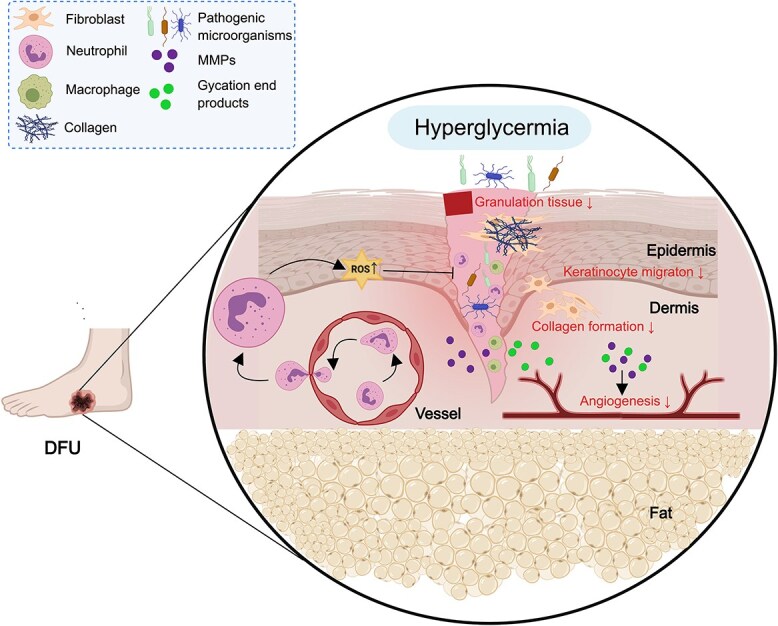
Diabetic wound healing process. Diabetic wounds are characterized by severe oxidative stress, impaired angiogenesis and matrix turnover, and external damage, such as microbial infection. Angiogenesis is inhibited by the accumulation of MMPs and advanced glycation end products, thereby delaying diabetic wound closure (Created with MedPeer). *MMPs* matrix metalloproteinases, *DFU* diabetic foot ulcer, *ROS* reactive oxygen species

The fundamental evolutionary impetus in cutaneous injuries that heal quickly and do not cause an underlying pathophysiological abnormality may be to achieve healing swiftly and with the least amount of energy [31]. Chronic nonhealing wounds do not progress through the aforementioned linear phase of overlapping biological processes [32,33]. Diabetes impairs the finely controlled course of the healing process [34]. In wounds with diabetic ulcers, evolutionary adaptations are unlikely to occur, resulting in poor healing [24]. Chronic wounds pose several obstacles for treatment, including hypoxia, necrotic tissues, poor pharmacokinetics, and cellular abnormalities [33]. For example, increased glucose levels inhibit M1 to M2 macrophage conversion, causing a decrease in the number of myofibroblasts, insufficient collagen release, and delayed wound closure [26]. Several pathological problems contribute to the failure to heal, including inherent DM-specific defects in the blood supply, angiogenesis, and matrix turnover, as well as external defects induced by infection, biofilm formation or repetitive damage [31]. Diabetes-related traumatic skin tissues are subjected to more severe oxidative stress than normal wound skin tissues are. High levels of oxidative stress damage DNA, proteins, and lipids within cells, ultimately resulting in cell death and consequent tissue malfunction [35]. Absolute or relative insulin insufficiency causes a rise in glucose and a disruption in lipid metabolism. Deviations from normal energy metabolism may result in aberrant immune cells and signal transmission, both of which are important for the chronic wound healing process [36]. Over time, cytokine production and receptor expression are compromised in a hyperglycemic environment, impairing macrophage and other cell functions [34,37]. Angiogenesis is hampered by the buildup of matrix metalloproteinases (MMPs) and advanced glycation end products, thereby delaying diabetic wound closure [38]. In addition, a range of pathways that lead to neuropathy, including the protein kinase C pathway, hexosamine pathway, polyol pathway, diacylglycerol pathway, and nitric oxidase synthase pathway, hinder diabetic wound healing [39] (Figure 2).

Peripheral artery dysfunction, diabetic neuropathy, and local infection are widely accepted as the primary explanations for diabetic ulcers [40]. For example, chrysin counteracts the inhibitory effect of EC-derived exosomal miR-92a on Kruppel-like factor 2 (KLF2) expression to protect blood arteries from atherosclerosis [41]. Via the miR-513a-5p/TGFBR3 competing endogenous RNA network pathway, exosome-derived circ0001785 reduces EC damage and delays atherogenesis [42]. In addition, electroconductive hydrogels (ECHs) loaded with BMSC-exos (ECH-exos) may facilitate Schwann cell adhesion and migration. ECH-exos also reduced inflammatory pain by modifying the polarization of M2 macrophages via the NF-κB pathway. Through the MEK/ERK pathway, ECH-exos improved myelinated axonal regeneration, which in turn reduced muscle denervation atrophy and encouraged functional restoration [43]. The incorporation of BMSC-exos into a hyaluronan-collagen hydrogel (DHC-BME) stimulated angiogenesis and neurogenesis, which together supported axonal regeneration, synaptogenesis, neuronal differentiation, and even structural remodeling in the brain [44]. In diabetic patients suffering from peripheral nerve damage and vascular injury, exosomes have great potential for stimulating vascularization, nerve regeneration, and functional restoration.

### Mechanisms of exosomes in diabetic wound healing

**Figure 3:**
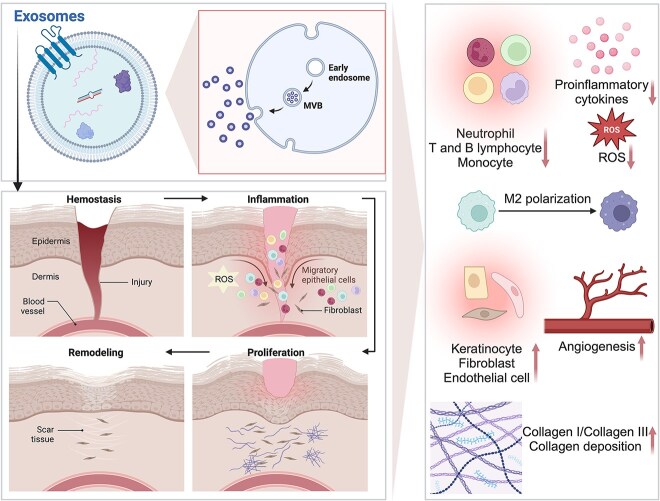
Effects of exosomes on diabetic wound healing. Exos can alter the biology of wound healing at each stage of diabetic wound healing. They participate in modulating the inflammatory response, accelerating proliferation and reepithelization, increasing angiogenesis, and regulating ECM remodeling (Created in BioRender). *MVB* multivesicular body, *ROS* reactive oxygen species

Exos can alter the biology of wound healing at each stage by either promoting or inhibiting certain bioactivities relevant to diabetic wound healing (Figure 3).

#### Regulating inflammation

Inflammation is the defense mechanism of the body in response to potentially hazardous stimuli. When platelets degranulate, the complement cascade is triggered, stimulating inflammatory cells and attempting to eradicate cell debris and pathogens in the wound region [45,46]. Gradually, macrophages play the role of neutrophils, and the phenotype of macrophages also changes as apoptotic cells are cleared [34]. On the other hand, prolonged inflammation is harmful and may lead to dysregulated activation and differentiation of keratinocytes, hindering wound healing progression through the usual phases [47]. To avoid excessive inflammation, macrophages polarize into the anti-inflammatory M2 phenotype [29]. Precise and appropriate cellular reactions to inflammatory mediators, growth factors, and mechanical stresses are required [31].

By modulating SIRT3/SOD2, ADSC-exos may alleviate inflammation, oxidative stress, and mitochondrial dysfunction, hence enhancing diabetic wound healing [48]. Menstrual blood-derived mesenchymal stem cell (MenSC)-derived exosomes can alleviate inflammation by polarizing M1 macrophages into M2 macrophages [49]. Moreover, epidermal stem cell (ESC)-derived exosomes (ESC-exos) enhance macrophage proliferation and migration and increase the number of M2 macrophages, thereby reducing inflammation in diabetic mice. The miRNAs of ESC-exos also demonstrated the regulatory control of TGF-β signaling pathways *in vitro* [50]. Additionally, in a diabetic rat model, exosomes produced from macrophages displayed anti-inflammatory effects by suppressing the release of proinflammatory cytokines and enzymes [51]. Fibrocyte-derived exosomes were shown to contain anti-inflammatory miRNAs (miR124a and miR-125b), accelerating wound repair in diabetic mice [52]. Inimitably, *Periplaneta americana* L. (PA)-derived exosome-like nanoparticles (PAELNs) were implicated in anti-inflammatory and reepithelialization mechanisms, and topical treatment with PAELNs might significantly accelerate wound closure in a diabetic mouse model [53]. According to these studies, exosomes can suppress the onset of excessive inflammation and mitigate its negative consequences on diabetic wounds, thereby improving diabetic wound healing.

#### Accelerating proliferation and reepithelialization

Fibroblasts multiply in the proliferative phase to create the ECM. Reepithelialization occurs when epithelial cells proliferate and move to the wound center, eventually covering the lesion entirely.

The MSC-derived exosomal lncRNA H19 inhibited miR-152-3p-mediated PTEN suppression and thereby prevented inflammation and fibroblast apoptosis in a mouse model of DFU [54]. In an *in vitro* coculture model and an *in vivo* diabetic mouse model, treatment with miR-155 inhibitor-loaded MSC-exos improved keratinocyte migration, expedited reepithelialization, and restored fibroblast growth factor-7 (FGF-7) levels [55]. In addition, ESC-exos promoted the viability of fibroblasts *in vitro* and augmented cell proliferation in wounds of db/db mice [50]. By stimulating keratinocyte and fibroblast migration and proliferation, ADSC-exos accelerated the healing process via activation of the heat shock protein 90 (HSP90)/LRP1/AKT signaling pathway in streptozotocin (STZ)-induced diabetic C57BL/6 mice and the TGF-β/Smad3 signaling pathway in B6.Lepr^db/db^ mice [56,57]. Another way through which exosomes can affect wound healing has been observed in exosomes produced from adipocyte progenitor cells. By delivering lncH19 to wounded tissue, these exosomes effectively reversed defective diabetes wound healing. Mechanistically, lncH19 alleviated fibroblast cycle arrest and promoted macrophage infiltration into injured tissues via the inhibition of GDF15 release and p53 activity [58]. Moreover, *in vitro* bioinformatic analysis revealed that BMSC-derived EVs had favorable effects on fibroblasts and keratinocytes, two types of cells involved in diabetic wound healing [14]. In addition, fibrocyte-derived exosomes activated diabetic dermal fibroblasts and keratinocytes *in vitro* and accelerated wound closure in B6.Lepr^db/db^ mice [52]. In summary, these findings indicate that exosomes have substantial potential to improve diabetic wound healing by encouraging reepithelialization.

#### Promotion of angiogenesis

Angiogenesis is a crucial process in wound repair. Angiogenesis is triggered by angiogenic factors such as vascular endothelial growth factor (VEGF) and FGF-2, which are generated by fibroblasts, keratinocytes, and inflammatory cells [59]. Neovascularization facilitates the flow of nutrients and waste products from metabolism and promotes tissue repair by supplying blood for wound healing [34].

In an environment with high glucose (HG), ADSC-exos stimulate the proliferation and angiopoiesis of endothelial progenitor cells (EPCs). ADSC-exos overexpressing Nrf2 resulted in increased angiogenesis, granulation tissue development, and growth factor expression along with decreased levels of oxidative stress-related proteins in diabetic rat model wound beds [60]. ADSC-exos also promoted periwound vascularization and expedited wound healing by reducing reactive oxygen species (ROS) formation in human umbilical vein endothelial cells (HUVECs) and the amount of oxidative stress caused by excessive hyperglycemia [48]. In addition to reducing ROS levels, ADSC-exos might enhance EC proliferation and neovascularization via the HSP90/LRP1/AKT signaling pathway, thereby promoting diabetic wound healing *in vivo* [56]. Analyses using *in vitro* bioinformatics revealed that ADSC-EVs exerted beneficial effects on ECs involved in diabetic wound repair [14]. Wang et al. reported that ESC-exos stimulated angiogenesis and promoted wound closure in db/db mice [50]. Furthermore, ESC-EVs loaded with VH298 (VH-EVs) and bioinspired nanovesicles (NVs) loaded with dapagliflozin from induced pluripotent stem cell-derived ECs enhanced wound repair in diabetic mice by locally increasing angiogenesis and blood flow, which was likely associated with the activation of the hypoxia inducible factor-1α (HIF-1α)/vascular endothelial growth factor A (VEGFA) axis [61,62]. Han et al. reported that the BMSC-derived exosomal lncRNA KLF3-AS1 suppressed apoptosis while enhancing HUVEC proliferation and migration in an HG milieu. KLF3-AS1 accelerated cutaneous wound healing *in vivo* partially through the miR-383/VEGFA signaling pathway [63]. Chen et al. demonstrated that exosomes from human urine-derived stem cells (USC-exos) might promote diabetic wound healing by enhancing angiogenic responses of ECs as well as angiogenesis, which requires the deleted in malignant brain tumors 1 (DMBT1) protein [64]. In addition, human umbilical cord mesenchymal stem cell (HUCMSC)-derived exosomes (HUC-exos) enhanced angiogenesis and expedited the wound healing process in diabetes by alleviating oxidative stress in ECs [65].

Yu et al. reported that exosomal miR-221-3p from EPCs enhanced capillary-like tube formation and HUVEC vitality, as well as wound closure, in diabetic mice by suppressing HIPK2 [66]. On the other hand, exo-mediated LINC01435 taken up by HUVECs cooperates with the transcription factor Yin Yang 1 (YY1) to upregulate histone deacetylase (HDAC)8 expression, thus suppressing angiogenesis [67]. Additionally, macrophage-derived exosomes stimulate endothelial cell migration and proliferation to facilitate angiogenesis, which expedites the healing process [51]. Exos produced from fibrocytes were loaded with proangiogenic miRNAs (miR-126, miR-130a, and miR-132) and promoted wound closure in diabetic mice [52]. Nearly every bodily fluid, such as blood, breast milk, semen, urine, bile, saliva, and amniotic fluid, contains exosomes [33]. Milk exosomes loaded with miR-31-5p enhanced EC activities *in vitro*, angiogenesis and diabetic wound repair *in vivo* [68]. Xiang et al. also demonstrated that milk-derived exosomes combined with siKeap1 could relieve oxidative stress in HUVECs and promote diabetic wound repair with neovascularization in a diabetic mouse model [69]. In addition to milk, Chen et al. revealed that serum exosomes (serum-exos) enhanced tube formation in HUVECs and facilitated angiogenesis and subsequent wound repair in diabetic mice [70]. However, miR-15a-3p was demonstrated to be overexpressed in exosomes from the blood of diabetes patients, impairing wound healing. Xiong et al. reported that inhibiting circulating exosomal miR-15a-3p expedited the healing of diabetic wounds via NOX5 activation [71]. Collectively, these findings point to the remarkable potential of exosomes to facilitate angiogenesis and, consequently, the wound healing process in diabetes patients (Table 1).

**Table 1:** Exosomes in animal models that accelerate angiogenesis in diabetic wound healing

**Wound type**	**Model**	**Interventions and dose**	**Source**	**Results**	**Reference**
A round full-thickness skin wound	Rat	Skin defects in a diabetic rat model were treated with EPCs alone or combined with ADSC-exos or ADSC-exos overexpressing Nrf2 for 0, 7, and 14 days postoperatively.	ADSC	In a high glucose environment, ADSC-exos enhanced proliferation and angiopoiesis in EPCs, and Nrf2 overexpression strengthened this protective effect.	[56]
A round full-thickness skin wound	Mice	Subcutaneous injection of PBS or ADSC-exos (200 μg)	ADSC	ADSC-exos enhanced periwound vascularization by lowering HG-induced oxidative stress levels.	[48]
A round full-thickness skin wound	Mice	Subcutaneous injection of ADSC-exos (50 μg per mouse) and PBS (equal volume to the ADSC-exos suspension); divided into Nor + PBS, Dia + PBS, Dia + exos, Dia + exos + IgG, and Dia + exos + anti-HSP90	ADSC	ADSC-exos accelerated endothelial cells proliferation and neovascularization, and lower ROS levels via HSP90/LRP1/AKT signaling pathway.	[56]
Two circular full-thickness wounds	Mice	Subcutaneous injection of ESCs or exosomes diluted to 5 × 10^6^/ml or 50 μg/ml in PBS	ESC	ESC-exos stimulated angiogenesis and hastened diabetic wound healing.	[50]
Two full-thickness skin wounds	Mice	Micro syringe injection; divided into 100 μl PBS solution, VH298 (20.34 μM), EVs (2 × 10^9^ particles/ml), and VH-EVs (20.34 μM, 2 × 10^9^ particles/ml)	ESC	VH-EVs locally enhanced blood flow and angiogenesis, which was possibly associated with HIF-1 α/VEGFA signaling pathway.	[62]
Rectangular full-thickness wound	Mice	Tail vein injection of 100 μl OE-NC-exos, OE-KLF3-AS1-exos, shNC-exos or shKLF3-AS1-exos	BMSC	LncRNA KLF3-AS1 from BMSC-derived exosomes (BMSC-exos) enhanced proliferation of HUVECs in HG milieu and accelerated cutaneous wound healing *in vivo* partially through miR-383/VEGFA signaling pathway.	[63]
Two full-thickness excisional skin wounds	Mice	Subcutaneously injection; divided into PBS group (100 μl PBS), USCs^Con shRNA^-exos group (200 μg USCs^Con shRNA^-exos in 100 μl PBS), USCs^shDMBT1 #1^-exos group (200 μg USCs^shDMBT1 #1^-exos in 100 μl PBS)	USC	USC-exos might increase angiogenic responses of ECs, and angiogenesis via DMBT1 protein.	[64]
Full-thickness excision skin wounds	Mice	Injection of PBS (100 ul), HUC-exos (50 μg/ml), and HUC-exos (100 μg/ml)	HUCMSC	HUC-exos enhanced angiogenesis and accelerated healing process of diabetic wounds by alleviating oxidative stress in endothelial cells.	[65]
Full-thickness skin wounds	Mice	Subcutaneous injection of miR-221-3p agomir (2.5 nmol/wound) and a negative control (NC)	EPC	EPC-derived exosomal miR-221-3p promoted HUVEC vitality *in vitro* and wound repair process in diabetic mice via HIPK2 inhibition.	[66]
A round full-thickness skin wound	Mice	Subcutaneously injection of NG-exos or HG-exos (100 μg dissolved in 100 μl PBS) and a control group (25 μl PBS)	HUVEC	Exo-mediated LINC01435 uptake in HUVECs collaborated with YY1 to upregulate HDAC8 expression, leading to reduced tube formation, HUVECs migration.	[67]
Round full-thickness excision skin wounds	Rat	Subcutaneous injection of 1 ml PBS, 1 ml of low-concentration exos (100 μg/ml), high-concentration exos (1 mg/ml) and the combination of high-concentration exos (1 mg/ml) and LPS (10 μg/ml)	Macrophage	Macrophage-derived exosomes enhanced functions of ECs and angiogenesis, hence speeding up diabetic wound healing.	[51]
Two full-thickness excisional wounds	Mice	Subcutaneous injection and directly applied 200 μl of PBS containing 0, 5 or 50 μg exosome proteins	Fibrocyte	Fibrocyte-derived exosomes were loaded with proangiogenic miRNAs (miR-126, miR-130a, and miR-132) and promoted wound closure in diabetes.	[52]
A full-thickness excision wound	Mice	Divided into PBS (control) group, mimic NC group (2 nmol/wound), free miR-31-5p mimic group (2 nmol/wound), mEXO group (1.0 μg/μl), mEXO-NC group (1.0 μg/μl) and mEXO-31 group (1.0 μg/μl)	Milk	MiR-31-5p-exosomal formulation enhanced ECs activities *in vitro*, and improved angiogenesis and diabetic wound repair *in vivo*.	[68]
Full-thickness excisional wounds	Mice	Divided into PBS (STZ control) group, mEXOs (2 μg/wound), siKeap1 (2 μg/wound), mEXOs-siKeap1 (2 μg/wound), and PBS (normal control) group	Milk	Milk derived exosomes combined with siKeap1 could enhance HUVECs vitalities and promote diabetic wound repair with neovascularization *in vivo*.	[69]
Two round second degree scald wounds	Mice	Divided into normal group (intraperitoneal injection of citrate buffer), diabetic mice vehicle group (subcutaneously injected of 100 μl PBS) and diabetic mice serum-exos group (subcutaneously injected of 100 μg serum-exos in 100 μl PBS)	Serum	Serum-exos sped up angiogenesis by enhancing tube formation in HUVECs and subsequent wound healing *in vivo*.	[70]
A full-thickness excisional wound	Mice	Divided into Control group (100 μl PBS), Con-exos group (200 μg Con-exos in 100 μl PBS), Dia-exos group (200 μg Dia-exos in 100 μl PBS), AntagomiR-15a-3p group (2 OD antagomiR-15a-3p in 100 μl diethyl pyrocarbonate-treated water), and Dia-exos^antagomiR-15a-3p^ group (2 OD antagomiR-15a-3p in 100 μl diethyl pyrocarbonate-treated water and 200 μg Dia-exos in 100 μl PBS)	Blood	Inhibiting circulating exosomal miR-15a-3p restored HUVECs function and accelerated diabetic wound healing via NOX5 activation.	[71]

*EPCs* endothelial progenitor cells, *ADSC* adipose mesenchymal stem cell, *ADSC-exos* ADSC-derived exosomes, *Nrf2* nuclear factor erythroid 2-related factor 2, *PBS* phosphate-buffered saline, *HG* high glucose, *HSP90* heat shock protein 90, *ROS* reactive oxygen species, *ESC* epidermal stem cell, *ESC-exos* ESC-derived exosomes, *EVs* extracellular vesicles, *VH-EVs* ESC-EVs loaded with VH298, *HIF-1α* hypoxia inducible factor-1α, *VEGFA* vascular endothelial growth factor A, *BMSC* bone marrow mesenchymal stem cell, *lncRNA* long noncoding RNA, *HUVECs* human umbilical vein endothelial cells, *USC* human urine-derived stem cell, *USC-exos* USC-derived exosomes, *ECs* endothelial cells, *DMBT1* deleted in malignant brain tumors 1, *HUCMSC* human umbilical cord mesenchymal stem cell, *HUC-exos* HUCMSC-derived exosomes, *YY1* transcription factor Yin Yang 1, *HDAC8* histone deacetylase 8

#### Improving tissue remodeling

Matrix remodeling is associated with tissue reorganization. Type I collagen progressively replaces type III collagen as the tissue remodels, increasing strength and flexibility [30]. After a wound heals, scarring is a histological and morphological alteration of the skin tissues that affects appearance and compromises organ function [4].

Ren et al. demonstrated that ADSC-exos might hasten diabetic wound closure by stimulating collagen deposition through the HSP90/LRP1/AKT axis [56]. Moreover, exosomes from human adipose-derived mesenchymal stem cells (hADSC-exos) increased the synthesis of collagen in a mouse model of diabetes, and the inhibition of MMP1 and MMP3 strengthened their effect [72]. Gondaliya et al. reported that treatment of MSC-exos with miR-155 inhibitors resulted in increased collagen deposition, which accelerated diabetic wound repair *in vivo* [55]. Geiger et al. revealed that exosomes produced from fibrocytes were incorporated with miR-21, a miRNA that regulates collagen deposition and sped up wound closure in diabetes [52]. In addition, serum-exos can increase collagen-α and fibronectin expression, facilitating ECM formation in diabetic wound healing [70]. Taken together, these studies indicate that exosomes can influence collagen deposition and ECM remodeling, hence enhancing the wound healing process in patients with diabetes.

### Applications of exosomes in diabetic wound healing

#### Delivery of exosomes to diabetic wounds

The main issue with the healing process for diabetic wounds is that the temporal axis of wound restoration is thrown off. There are still unresolved issues with hypoxia, immune cell dysfunction, bacterial growth, and biofilm formation [73]. Four types of drug delivery systems are available for treating skin wounds: spraying, local injection, use in conjunction with biomaterials, and systemic treatment [74]. In addition to maximizing the function of exosomes themselves, improved delivery strategies are needed to support the therapeutic effects of exosomes [75]. Scaffolds are the best materials for preserving, enhancing, and regaining tissue function, according to the definition of tissue engineering given by the National Science Foundation [76]. Dressings are more likely to provide a healing environment than are scaffolds, even though scaffolds are more likely to operate as conduits and storage materials for active chemicals. They can optimize the function of growth factors and endogenous cells and, as a result, transport certain medications or inorganic chemicals, allowing for the versatile use of biomaterial-based wound healing materials.

##### Exos in conjunction with hydrogels or dressings

Exos can aid in the healing of diabetic wounds. Although exosomes can enhance skin regeneration, designing and selecting an optimum way to administer exosomes has been regarded as a major difficulty. Exos clear quickly, but their distribution has extremely limited penetration across the stratum [77]. Hydrogels can facilitate gas exchange, supply vital nutrients, govern cell migration and proliferation, and distribute bioactive chemicals during wound healing [78]. They can be utilized as scaffolding materials to collect exosomes and stimulate wound healing in a synergistic manner [79,80].

A new *in situ* injectable HA@MnO2/FGF-2/Exos hydrogel covered the wounds in a layer of protection, allowing for oxidative stress reduction and long-term antibacterial protection, hence enhancing the healing of diabetic wounds in mice. Furthermore, M2 macrophage-derived exosomes (MEs) and FGF-2 promote reepithelization and angiogenesis, respectively [81]. Wang et al. reported that the FHE hydrogel (F127/OHA-EPL) exhibited multiple functions, such as effective antibacterial activity, prolonged release of pH-responsive bioactive exosomes, and rapid self-healing. The FHE@exosomes (FHE@exos) hydrogel markedly increased the capacity of HUVECs to form tubes *in vitro* and improved the *in vivo* healing efficiency of diabetic wounds [78]. Another hydrogel with sustained pH-responsive exosome release ability is an injectable thermosensitive polysaccharide-based dressing (FEP). FEP@exosomes (FEP@exos) markedly promoted the ability of ECs to migrate, proliferate, and form tubes *in vitro*. FEP@exos accelerated angiogenesis to expedite wound repair in diabetic mice [82]. Moreover, an ADSC-exo@MMP-PEG hydrogel and ADSC-exos loaded in an alginate hydrogel have been shown to contribute to diabetic wound healing *in vivo* [83,84]. Hypoxia-pretreated ADSC-exos (ADSC-HExos)-embedded methacrylate gelatine (GelMA) hydrogels (GelMA-HExos) exhibited porosity and a steady rate of expansion and degradation in vitro. In diabetic mice, circSnhg11 administration via GelMA-HExos hydrogels may enhance wound healing [85]. Yang et al. revealed that combining Pluronic F-127 (PF-127) with HUC-exos enhanced granulation tissue regeneration and elevated the levels of CD31, Ki67, TGFβ-1, and VEGF. They demonstrated that HUC-exos in a PF-127 gel considerably accelerated wound closure in diabetic rats [86]. HUC-exos encapsulated in polyvinyl alcohol (PVA)/alginate nanohydrogel (exo@H) facilitated HUVEC viability and the healing of diabetic wounds. Zhang et al. confirmed that exo@H augmented VEGF levels through the ERK1/2 pathway and elevated SR-B1, SMA, and CD31 expression [87]. In addition, bioinspired adaptive indwelling microneedles (MNs), which consist of MSC-exos encapsulating adjustable PVA hydrogel needle tips and detachable 3 M medical tape supporting substrates, have been created for the treatment of diabetic wounds. These indwelling MNs facilitated tissue regeneration in diabetic rat models [88]. To investigate drug-bioactive substance combinations, a PEG/Ag/CNT-M + E hydrogel, a highly interconnected porous network that can mobilize and release metformin and exosomes, was created. It aids in wound repair by stimulating proliferative and angiogenetic ability while also alleviating vascular damage and inflammation [89]. GelMA-dopamine-EVs restored the homeostasis of the healing milieu of diabetic wounds, accelerating wound closure and encouraging the restoration of skin structure in rats [90]. Yuan et al. reported an MN patch consisting of a GelMA/PEGDA hydrogel to accomplish the regulated release of exosomes and tazarotene. The GelMA/PEGDA@T + exos MN patch improved the ability of cells to proliferate and generate blood vessels both *in vitro* and *in vivo* [91]. Furthermore, Yu et al. successfully incorporated ECM with VEGF-encapsulated activated polymorphonuclear neutrophil (PMN) exosome mimetics (aPMNEMs) to create a VEGF–aPMNEM–ECM hydrogel for the treatment of diabetic wounds [92]. In addition, a GelMA hydrogel containing VH-EVs (Gel-VH-EVs) improved angiogenesis and blood flow via the HIF-1α/VEGFA signaling pathway to promote wound closure in diabetic mice [62].

OxOBands, porous cryogels with sustained oxygen release capabilities, induce the antioxidant polyurethane (PUAO) and ADSC-exos. OxOBands accelerated reepithelialization, neovascularization, and collagen deposition while reducing oxidative stress in diabetic wounds. OxOBands also stimulate the growth of mature epithelial structures comparable to those in healthy skin [93]. ADSC-exos incorporated into a scaffold of the human acellular amniotic membrane (hAAM) facilitated the activities of human dermal fibroblasts (HDFs) and HUVECs. The hAAM-exo dressing also facilitated wound repair in a diabetic mouse model [94]. Moreover, a sprayable alginate hydrogel (SA) dressing that contains exosomes and oxygen-producing microspheres promoted local oxygen production and accelerated full-thickness wound healing characteristics [95]. Zeng et al. presented a double-layer MN-based wound dressing system (MEs@PMN) that encapsulated MEs in needle tips and contained polydopamine (PDA) nanoparticles in the backing layer. MEs enhance macrophage polarization towards the M2 phenotype, and the photothermal effects of MEs and PMNs generate a combination of proangiogenic effects by increasing von Willebrand factor (vWF) and CD31 expression [96]. Therefore, MEs@PMN had a beneficial effect on diabetic wound repair. Taken together, these results offer a novel bioactive approach for treating diabetic wounds and point to a viable combination strategy based on exosomes (Table 2).

**Table 2:** Exosomes complexed with hydrogels or dressings for diabetic wound healing

**Hydrogel or dressing**	**Exosome source**	**Model**	**Results**	**Reference**
HA@MnO2/FGF-2/Exos hydrogel	M2 macrophage	Mice	HA@MnO2/FGF-2/Exos hydrogel generated a suitable milieu for activation of angiogenesis and epithelization, resulting in significantly better wound closure in diabetes.	[81]
FHE@exos hydrogel	ADSC	Mice	FHE@exos hydrogel dramatically enhanced HUVEC vitality and angiogenesis. In vivo, FHE@exos hydrogel also stimulated cellular proliferation and neovascularization, hence hastening the healing process.	[78]
FEP@exos hydrogel	ADSC	Mice	FEP@exos enhanced diabetic wound healing by improving proliferative and angiogenetic abilities in regenerated tissue, resulting in quicker granulation tissue development and collagen deposition.	[82]
ADSC-exo@MMP-PEG hydrogel	ADSC	Mice	ADSC-exo@MMP-PEG treatment considerably ameliorated the oxidative stress induced by H2O2 and promoted diabetic wound repair *in vivo*.	[83]
Alginate-based hydrogel	ADSC	Rat	ADSC-exos with alginate-based hydrogel enhanced collagen synthesis and tube formation in the diabetic wound area.	[84]
GelMA-HExos hydrogel	ADSC	Mice	CircSnhg11 delivery by GelMA-HExos hydrogels could promote angiogenesis and diabetic wound repair *in vivo*.	[85]
PEG/Ag/CNT-M + E hydrogel	ADSC	Mice	PEG/Ag/CNT-M + E hydrogel could release metformin and exosomes, which stimulated cell proliferation and angiogenesis while alleviating vascular damage and inflammation.	[89]
PF-127 gel	HUCMSC	Rat	HUC-exos in PF-127 gel drastically upregulated the expression of CD31, Ki67, VEGF, and TGFβ-1, stimulating diabetic wound repair.	[86]
Exo@H	HUCMSC	Rat	Exo@H promoted HUVECs function and healing efficiency of diabetic wounds by augmenting the production of angiogenesis-related molecules and activation of ERK1/2 pathway.	[87]
Bioinspired adaptable indwelling microneedles	MSC	Rat	Indwelling microneedles promoted tissue regeneration in diabetic wounds by successfully activating macrophages, fibroblasts, and vascular endothelial cells.	[88]
GelMA-dopamine hydrogel	MSC	Rat	MSC-EVs and the GelMA-dopamine hydrogel worked together to restore the homeostasis of the healing milieu of diabetic wounds, which sped up wound closure.	[90]
GelMA/PEGDA@T + exos MNs patch	HUVEC	Mice	GelMA/PEGDA@T + exos MNs patch could regulate the release of exos and tazarotene, and enhance proliferation and angiogenesis process, thus hastening diabetic wound healing.	[91]
VEGF–aPMNEM–ECM hydrogel	PMN	Rat	VEGF–aPMNEM–ECM could limit bacterial growth, encourage tube formation, polarize macrophages in the wound area, and effectively aid in *in vivo* wound healing.	[92]
Gel-VH-EVs	ESC	Mice	Gel-VH-EVs improved blood supply as well as angiogenesis *in vivo* via activation of HIF-1α/VEGFA signaling pathway.	[62]
OxOBand	ADSC	Rat	OxOBand reduced infection and ulceration, and facilitated re-epithelialization and collagen deposition in clinically problematic infected diabetic wound.	[93]
hAAM-exos dressing	ADSC	Mice	The hAAM-exos dressing improved the healing rate of diabetic wound through the regulation of inflammation, vascularization, and ECM remodeling.	[94]
CP/EXO/SA dressing	BMSC	Rat	CP/EXO/SA dressing appeared to hasten the healing of full-thickness diabetic wounds, resulting in favorable collagen deposition, re-epithelization, and angiogenesis at the wound beds.	[95]
MEs@PMN	M2 macrophage	Rat	MEs@PMN suppressed inflammation while improving angiogenesis both *in vitro* and *in vivo* to speed up wound closure of diabetes.	[96]
SP@PRP-Exos	MSC	Rat	Dual-crosslinked hydrogels facilitated quicker diabetic wound healing by downregulating the expression of MMP-9 and stimulating angiogenesis and re-epithelialization.	[97]

*HA* hyaluronic acid, *FGF-2* fibroblast growth factor 2, *FHE* F127/OHA-EPL, *ADSC* adipose mesenchymal stem cell, *HUVEC* human umbilical vein endothelial cell, *ADSC-exos* ADSC-derived exosomes, *ADSC-HExos* hypoxia-pretreated ADSC-exos, *GelMA* methacrylate gelatine, *HUCMSC* human umbilical cord mesenchymal stem cell, *HUC-exos* HUCMSC-derived exosomes, *PF-127* Pluronic F-127, *VEGF* vascular endothelial growth factor, *TGFβ-1* transforming growth factor beta-1, *exo@H* HUC-exos encapsulated in polyvinyl alcohol/alginate nanohydrogel, *MSC* mesenchymal stem cells, *MSC-EVs* MSC-derived extracellular vesicles, *MNs* microneedles, *PMN* polymorphonuclear neutrophil, *ESC* epidermal stem cell, *HIF-1α* hypoxia inducible factor-1α, *VEGFA* vascular endothelial growth factor A, *hAAM* human acellular amniotic membrane, *BMSC* bone marrow mesenchymal stem cell, *MMP-9* matrix metalloproteinase-9

Dual-crosslinked hydrogels can overcome the limitations of single ingredients and may lead to the development of a novel class of diabetic wound dressings. On the basis of silk protein (SP) (sericin and fibroin), Bakadia et al. developed three types of dual-crosslinked hydrogels: SP@MSC-Exos, SP@platelet-rich plasma (PRP), and SP@PRP-Exos. Compared with PRP and SP, the dual-crosslinked hydrogels facilitated diabetic wound healing *in vivo* via the downregulation of MMP-9 expression and promoted angiogenesis and reepithelialization [97]. Recently, reports on the function of MSCs combined with biomaterials in the repair of diabetic wounds have been published. Compared with commonly utilized protease-degradable hydrogels, hydrolytic hydrogels break down at rates that allow the cutaneous wounds of diabetic model mice to heal without hindrance while also improving the local persistence of MSCs [98]. In combination with metformin, the MSC/fibrin scaffold (FG) recruited keratinocytes and fibroblasts, which in turn stimulated migration and VEGF-mediated angiogenesis in an Akt/mTOR-dependent manner during diabetic wound healing [99]. After being wrapped in a silk fibroin chitosan film coated with ADSC, the wound tissue almost fully regenerated at a location near normal tissue [100]. Sun et al. reported a new skin ECM-biomimetic coaxial nanofibrous scaffold to dynamically modify the microenvironment for the healing of diabetic wounds. The improved M1-to-M2 macrophage polarization indicated that the biomimetic coaxial scaffolds significantly increased ADSC immunomodulatory paracrine secretion [101]. These newly reported applications of biomaterials or their combination with cells in diabetic wounds may have the potential to be expanded to include exosomes.

##### Potential directions for incorporating biomaterials

Three-dimensional (3D) scaffolds may be tailored to accommodate a range of diabetes wounds because of their controlled structure and alignment. Li et al. demonstrated that a nanofiber/hydrogel core shell scaffold with a 3D multilayer patterned structure (3D-PT-P/GM) could efficiently create a moist environment and absorb exudate, which significantly accelerated the healing process of diabetic wounds with enhanced formation of a 3D capillary network [102]. The 3D cavity-structured hADSC spheroid delivery system improved the cell homing effect and level of therapeutic growth factor at the wound site, enhancing the wound healing effect [103]. MSC-loaded 3D nanofiber scaffolds can augment granulation tissue development, reduce inflammation, and facilitate angiogenesis and collagen deposition in type 2 diabetic wounds [104]. Letizia et al. employed 3D-printed patches to deliver MSC-EVs using a bioink made of methacrylated hyaluronic acid (MeHA). By permitting the regulated release of MSC-EVs, the MeHA patch increased wound epithelialization, angiogenesis, and innervation in a diabetic ulcer pressure model [105]. In addition, 2D carbide (MXene)-ME nanohybrids (FM-exos) sustainably released MEs for up to 7 days with broad-spectrum antibacterial action. FM-exos can overcome the immunological suppression derived from HG levels and promote diabetic wound healing [106]. Taken together, 2D or 3D scaffolds may be better able to increase cellular activity and control the rate of exosomal release at wound sites.

Endothelial nitric oxide synthase (eNOS)-produced nitric oxide (NO) prevents platelets from adhering to vessel walls and enhances blood perfusion. In the wound-healing timeline, NO has also been demonstrated to increase MMP concentrations, coordinate the recruitment of leukocytes and keratinocytes, and lower bacterial loads [107–110]. NO controls epidermal permeability barrier homeostasis, while topical applications of a variety of NO donors, such as S-nitroso-N-acetyl-D,L-penicillamine (SNAP), can delay permeability barrier recovery in skin with disturbed barriers [111]. Recent research has shown that asiaticoside-NO promotes diabetic wound healing via the miRNA-21-5p/TGF-β1/SMAD7/TIMP3 and Wnt/β-catenin signaling pathways [112,113]. However, artificial application is highly challenging because of the short half-life, intense reactivity, and limited diffusion distance of NO [114]. A NO microbubble-capturing hydrogel (PNO) may promote the healing of DFU by reversing sustained inflammation and promoting angiogenesis [115]. Dharshan et al. reported that topical administration of NO-gel greatly promoted a more regenerative ECM architecture at diabetic wound sites, which resulted in randomly oriented collagen fibers similar to those in unwounded skin [116]. A polylactic acid nanofibrous layer loaded with NO-eluting nanoparticles enhanced angiogenesis, allowing diabetic wound sites to receive NO either exogenously or endogenously [117]. By encapsulating a NO donor and reducing silver nanoparticles (Ag) *in situ*, Huang et al. developed a sprayable nanogel (Ag-G@CS) based on chitosan. In diabetic wounds, Ag-G@CS cooperatively eliminates infection and biofilms, reduces inflammation, and increases VEGF [118]. Tu et al. crosslinked hydrophilic poly(PEGMA-co-GMA-co-AAm) (PPGA) polymers with hyperbranched poly-L-lysine (HBPL)-modified manganese dioxide (MnO2) nanozymes to prepare a multifunctional hydrogel, which was further loaded with pravastatin sodium to obtain the HMP hydrogel. Pravastatin sodium was intended to take part in the production of NO. The HBPL-crosslinked HMP hydrogel greatly accelerated diabetic wound closure during the inflammatory phase by efficiently treating the infection *in vivo* [119]. Zhou et al. combined an electrospun polycaprolactone (PCL) mat with a biomaterial that releases NO (CS-NO) to create a new functional wound dressing. Prolonged release of NO from PCL/CS-NO was shown to promote wound healing, including increased collagen production, angiogenesis, and immunomodulation [120]. Kavoos et al. loaded S-nitrosoglutathione (GSNO), a NO donor, into a carboxymethyl chitosan (CMC)/sodium alginate (ALg) composite film (CMC-ALg-GSNO). In contrast to CMC-ALg and gauze, the CMC-ALg-GSNO dressing facilitated wound healing in diabetic rats [121]. Moreover, the NO sustained-release hydrogel (CAT/bArg/GSON) combined adenine- and thymine-modified chitosan (CSA and CST) with GSNO and binary l-arginine (bArg). This injectable hydrogel dressing with dual NO donors improved blood vessel regeneration and bacterial eradication [122]. With a core-shell structure, copper-benzene-1,3,5-tricarboxylate (HKUST-1) has been shown to be a sustained NO release vehicle. The extra copper ions released from HKUST-1 act in concert with NO to improve anti-inflammatory properties, angiogenesis, and collagen deposition during the healing process of diabetic wounds [123]. Moreover, as the NO-loaded HKUST-1 metal–organic framework (MOF) was encapsulated with graphene oxide (GO), the resulting NO@HKUST-1@GO microparticles (NHGs) enabled photothermal-responsive NO administration to promote diabetic wound healing [124]. Xie et al. combined polyaniline (PANI) and GSNO with chitosan, polyvinyl alcohol, and hydroxypropyltrimethyl ammonium chloride chitosan (PVA/CS/HTCC) matrices and developed a photothermal dressing capable of releasing NO uniformly. Using this dressing facilitated a burst release of NO by near-infrared (NIR) irradiation, leading to the successful treatment of infected wounds in diabetic rats [125]. Similarly, to achieve mild photothermal antibacterial therapy (PTAT), a dynamic crosslinked hyaluronic acid (HA) hydrogel dressing (Gel-HAB) loaded with allomelanin (AMNP)-N, N′-dis-sec-butyl-N, N′-dinitroso-1,4-phenylenediamine (BNN6) nanoparticles (AMNP-BNN6) was capable of scavenging ROS and controllably releasing NO under NIR irradiation. Diabetic wounds tended to heal more quickly owing to the ability of the Gel-HAB hydrogel to effectively lower oxidative stress levels, manage infections, and promote angiogenesis [126]. In addition, glucose oxidase (GOx)-modified hyaluronic acid and L-arginine (L-Arg)-coupled chitosan were crosslinked *in situ* to create CAHG hydrogels. This allowed for the sustainable release of NO and hydrogen peroxide (H2O2) in the presence of a hyperglycemic environment. By suppressing bacteria, downregulating proinflammatory proteins, and increasing M2-type macrophages, angiogenesis and collagen deposition, H2O2 and NO released from CAHG hydrogels demonstrated greater efficacy for wound repair in a diabetic mouse model [127]. Since NO and oxygen have been demonstrated to expedite the healing process of diabetic wounds, Chen et al. described a hydrogel that reduced chronic inflammation and sped up neovascularization in diabetic mice by switching between the generation of oxygen and NO [128]. Li et al. created a hydrogel based on Ti3C2 MXene nanosheets coated with PDA and hyaluronic acid-graft-dopamine (HA-DA). This hydrogel was crosslinked by an oxyhemoglobin/hydrogen (HbO2/H2O2) system in conjunction with low photothermal stimulation to controllably release oxygen, accelerating infected diabetic wound closure [129]. Moreover, Cv-loaded microneedles (CvMN) can release oxygen in a controlled manner. They promoted angiogenesis and cell proliferation in diabetic mice, which successfully enhanced wound healing [130]. Asad et al. presented a hydrogel consisting of chitosan-polyvinyl alcohol (CS-PVA) and calcium peroxide (CPO), which are oxygen-releasing nanoparticles. This hydrogel was designed to provide oxygen gradually and continuously for at least five days, accelerating diabetic wound closure in rats [131]. Collectively, biomaterials can be loaded with gas to enhance their function in diabetic wounds. Biomaterials may carry both exosomes and gas to maximize the therapeutic effects. These findings offer a potentially more clinically transformative approach to diabetic wound healing.

Zhou et al. developed an intelligent NO nanogenerator triggered by acid decomposition of CaCO3. The pH-responsive release of NO can eliminate infection, promote vascular neogenesis, and increase the rate of diabetic wound closure [132]. Li et al. reported a pH-switchable glucose-initiated cascade reaction in a nanocomposite with several enzyme-like activities (Mo,Fe/Cu,I-Ag@GOx). As the wound pH changes the alkaline microenvironment, this nanocomposite breaks down H2O2 into O2 to lessen oxidative stress and relieve hypoxia [133]. Feng et al. combined an ALg hydrogel with a novel biodegradable copper hydrogen phosphate (CuP) nanozyme. The nanozymes released Cu ions continuously and exhibited NIR photothermal conversion capabilities and pH-responsive peroxidase/catalase-mimetic catalytic activity. These composite hydrogels enhanced angiogenesis in diabetic wounds in a high pH environment. NIR irradiation induces a moderate thermal effect that increases the bioactivity of Cu ions, which results in high antibacterial effectiveness in diabetic wounds [134]. The concept of wound dressing design may be expanded with the addition of external variables such as heat and visible light. In addition to the pH-responsive capacity of biomaterials, the synergistic combination of catalytic activity, photothermal effects, and released ions may provide tissue regeneration activity, making them extremely promising for various clinical applications. The integration of more intelligent biomaterials and exosomes has potential in diabetic wound applications. Furthermore, biological scaffolds or microneedles can improve the efficiency of wound healing by sustainably releasing exosomes. Productized biomaterials and exosomes are the way forwards.

Exos are difficult to administer accurately during systemic injection because of their half-life and the unstable wound environment, even though the ineffective vascular tissue of the wound may successfully distribute them. As a result, local intervention appears to be preferable for the treatment of diabetic wounds [135]. However, research has indicated that intravenous injections accelerate wound healing more so than localized injections do. This effect was thought to be attributed to the loss of exosomes following local injection and the disruption of the wound healing process by the syringe [136]. Additionally, according to some studies, exosomes obtained from individuals with diabetes show considerable increases in miRNA levels. These miRNAs may inhibit the angiogenesis of ECs by acting on several signaling pathways; thus, inhibiting circulating exosomal miRNAs may accelerate diabetic wound healing [74]. Moreover, Zhou et al. demonstrated that the combination of hADSC/hADSC-exo intravenous injection and local hADSC-exo smearing promoted wound healing via decreased scar formation and accelerated reepithelialization and angiogenesis. Considering the complex metabolic disorders in diabetic patients, exosomes may aid diabetic wound repair when applied both locally and systemically, but further study is needed.

#### Clinical applications of exosomes

According to the aforementioned studies, exosomes show considerable promise in diabetic wound healing. However, exosomes have a limited effect on the healing of wounds in diabetic patients. Jancy et al. conducted a first-in-human clinical study utilizing allogeneic platelet-derived EVs. Platelet EVs (pEVs) produced via the ligand-based exosome affinity purification (LEAP) procedure can be safely injected into people as a possible wound healing therapy [137]. Further investigations in clinical studies may be needed to determine its effectiveness in diabetic patients.

From clinicaltrials.gov, numerous trials have been conducted or are underway to assess the effectiveness, viability, and safety of exosome-based therapies for diabetic wound healing in humans. In diabetic patients with chronic skin ulcers (CCUs), one trial intends to evaluate the combined impact of MSC-exos and nutritional deficiency correction (NCT05243368). A trial on recruitment may contribute to personalized nutritional supplementation for healing and regenerative capacity. In addition, a phase 1 trial involved the use of conditioned media from Wharton’s jelly mesenchymal stem cells (WJ-MSCs) containing microvesicles or exosomes in CCUs (NCT04134676). Another trial in early phase 1 from Kumamoto University focused on the effect of autologous plasma-derived exosomes on refractory cutaneous ulcers (e.g. burns, decubitus, rheumatic disease, peripheral arterial disease, and chronic venous insufficiency) (NCT02565264). A phase 2 randomized controlled trial was initiated to evaluate the efficacy and safety of topically administered purified exosome product (PEP)-TISSEEL in patients with DFU (NCT06319287). In a recent clinical trial, adipose tissue exosome dressings combined with sterile hydrogels were administered to the wound surface (NCT05475418).

#### Current status of clinical application: Limitations and solutions

##### Limitations of exosomes in diabetic wounds

There are established models and techniques for creating wound healing model systems and confirming the efficacy of exosomes both *in vivo* and *in vitro* for fundamental research. Exos have been used in mouse, rat, rabbit, pig, Drosophila, and adult zebrafish models to treat diabetic wounds [138]. Importantly, animal models are utilized as antecedents to provide insights to assist subsequent human trials. The outcomes of these preclinical trials, however, do not correspond to those on human skin. Although the physiological structure of the skin of these species varies greatly from that of humans, only pig skin is similar to human skin [138,139]. Consequently, the bioeffects of exosomes on wound repair should be confirmed in a porcine model. Kua et al. reported that the administration of human umbilical cord lining epithelial cells (CLECs) to pig wounds encouraged faster healing without causing any negative side effects than did the use of human skin allografts (HSGs) [140]. However, porcine wound healing models still significantly differ from human wound healing models. This, along with their low cost and ease of handling, has made mice popular in the wound healing community [138]. In cases where human tissue samples are scarce, an alternative approach might involve the use of high-throughput cell biology technologies such as proteomics, metabolomics, or genomics to analyse samples for new mechanisms. Further studies and modifications might be performed in animal models for mechanisms that are comparable in human and animal wounds [139]. Currently, the poor concordance rate in animal models is a substantial impediment to clinically relevant studies. An appropriate preclinical model is still needed to accurately recapitulate human diabetic wound healing.

There are additional aspects that contribute to the complexity of a clinical wound in addition to its location, area, and diabetic wound environment. The participants’ inability to handle unforeseen circumstances and their conservative mindset are uncontrolled. Too many lost visits may necessitate the termination of several clinical trials. Different clinical studies have varying methods, outcomes, endpoint values, and follow-up times [141]. The lack of consistency and standardization complicates the comparison of the acquired data to other categories. Moreover, few studies have evaluated the adverse effects and the safety of exosomes has been given scant consideration. According to a meta-analysis of animal studies regarding EVs in wound healing, no adverse events, including skin morphology, renal or hepatic injury markers, immunologic function, or skin cell apoptosis, were reported in any of the studies [142]. The safety of the topical or systemic administration of exosomes in future clinical applications for the treatment of diabetic wounds requires more attention.

**Table 3:** Modifications of exosomes in diabetic wound healing

**Method of modification**	**Exosome source**	**Model**	**Results**	**Reference**
Hypoxic preconditioning	HUVEC	Mice	Hypoxic exosomes improved the expression of lncHAR1B and KLF4, which reduced EC dysfunction and promoted M2 macrophage differentiation under HG conditions.	[144]
Hypoxic preconditioning	ADSC	Mice	ADSC-HExos enhanced diabetic wound healing by regulating inflammation and ECM formation.	[145]
Hypoxic preconditioning	ADSC	Mice	GelMA-HExos sped up wound closure in diabetes by delivering circSnhg11 and enhancing angiogenesis.	[85]
Mmu_circ_0000250 modification	ADSC	Mice	Exosomes derived from mmu_circ_0000250-modified ADSCs inhibited apoptosis by activating autophagy and facilitated angiopoiesis in diabetic wounds.	[146]
Melatonin pretreatment	BMSC	Mice and Rat	MT-exos facilitated angiogenesis and collagen synthesis to aid diabetic wound healing.	[147]
Pioglitazone pretreatment	BMSC	Rat	PGZ-exos hastened diabetic wound repair by restoring HUVECs’ angiogenic function via the PI3K/AKT/eNOS pathway and promoting VEGF and CD31 expression and ECM remodeling.	[148]
Atorvastatin pretreatment	BMSC	Rat	ATV-exos improved activities and VEGF levels of ECs via the AKT/eNOS pathway and facilitated angiogenesis *in vivo*.	[149]
IFN-γ pretreatment	BMSC	Mice	IFN-γ-pretreated BMSC-derived exosomal miR126-3p exhibited angiogenetic efficacy in diabetic wounds via the SPRED1/Ras/Erk axis.	[150]
LPS pretreatment	HUCMSC	Rat	LPS pre-exos suppressed inflammation possibly through the let-7b/TLR4/NF-κB/STAT3/AKT axis and improved diabetic wound lesions.	[151]
High glucose preconditioning	HaCaT cell	Mice	HG-exos suppressed angiogenesis by the LINC01435/YY1/HDAC8 axis, which delayed the wound healing process.	[67]
Modified PMSCs transduced with SGM or pac-miR146a-pac protein	PMSC	Mice	Better than the SGM-miR146a-Exo-only and SFP-only treated groups, SGM-miR146a-Exo@SFP promoted wound healing linked to decreased neovascularization, inflammation, and collagen deposition.	[152]
TNF-α and hypoxic preconditioning	MSC	Mice	PCOF@E-exos might decrease oxidative damage and inflammation, enhance angiogenesis, and eliminate bacterial infection in a synergistic manner to accelerate wound closure.	[153]

*HUVEC* human umbilical vein endothelial cell, *EC* endothelial cell, *HG* high glucose, *ADSC* adipose mesenchymal stem cell, *ADSC-exos* ADSC-derived exosomes, *ADSC-HExos* hypoxia-pretreated ADSC-exos, *ECM* extracellular matrix, *GelMA* methacrylate gelatine, *BMSC* bone marrow mesenchymal stem cell, *MT-exos* melatonin-pretreated MSC-exos, *PGZ-exos* pioglitazone-pretreated MSC-exos, *eNOS* endothelial nitric oxide synthase, *VEGF* vascular endothelial growth factor, *ATV-exos* atorvastatin-pretreated MSC-exos, *IFN-γ* interferon-γ, *HUCMSC* human umbilical cord mesenchymal stem cell, *HG-exos* HG-pretreated immortalized human epidermal (HaCaT) cells, *YY1* transcription factor Yin Yang 1, *HDAC8* histone deacetylase 8, *PMSC* placental mesenchymal stem cell, *SGM* silk fibroin binding peptide-Gluc-MS2, *SFP* silk fibroin patch, *TNF-α* tumor necrosis factor-alpha

##### Solutions to dilemmas in clinical application

Exos have drawn much interest and effort because of their potential as a cell-free treatment for wound care as well as for diagnostic applications. To promote the therapeutic effects of exosomes in diabetic skin healing, new bioengineering approaches are being intensively investigated [32,75,143]. Exos might be modified by altering parent cells or directly designing exosomes for therapeutic agent encapsulation.

###### Modification of exosomes

Hypoxic exosomes increase lncHAR1B levels and subsequent KLF transcription factor 4 (KLF4) expression, alleviating EC dysfunction and driving the differentiation of M2 macrophages under HG conditions [144]. Wang et al. demonstrated that ADSC-HExos might suppress inflammation through the PI3K/AKT axis and enhance diabetic wound closure in a mouse model [145]. The aforementioned GelMA-HExos comprising ADSC-HExos and GelMA hydrogels could adapt to irregular diabetic wounds and expedite diabetic wound healing via circSnhg11 delivery [85]. Shi et al. reported that mmu_circ_0000250-modified ADSC-exos promoted autophagy activation by restoring EPC function under HG conditions. Modified ADSC-exos improved the wound healing process *in vivo* via the mmu_circ_0000250/miR-128-3p/SIRT1 axis [146]. Additionally, melatonin-pretreated MSC-exos (MT-exos) increased the ratio of M2-phenotype macrophages to M1-phenotype macrophages via the PTEN/AKT axis, which suppressed the proinflammatory factors TNF-α and IL-1β while increasing the level of the anti-inflammatory factor IL-10. MT-exos also inhibited inflammation *in vivo*, which considerably aided in the healing of diabetic lesions [147]. Hu et al. demonstrated that pioglitazone-pretreated MSC-exos (PGZ-exos) activated the PI3K/AKT/eNOS pathway to restore the viability of HUVECs injured by HG and increase angiogenesis to facilitate diabetic wound healing in a rat model [148]. Yu et al. revealed that atorvastatin-pretreated MSC-exos (ATV-exos) improved biological function and VEGF levels in ECs via the AKT/eNOS pathway *in vitro* and showed angiogenic capacity in diabetic skin defects *in vivo* [149]. Lu et al. transferred exosomal miR126-3p from interferon (IFN)-γ-pretreated BMSCs to HUVECs to accelerate diabetic wound healing, which enhanced angiogenesis via the SPRED1/Ras/Erk pathway [150]. In addition, LPS-preconditioned MSC-exos (LPS pre-exos) stimulated M2 macrophage activation and increased the expression of anti-inflammatory cytokines, possibly through the let-7b/TLR4/NF-κB/STAT3/AKT axis. In a diabetic rat model, LPS preexposure also considerably attenuated inflammation and aided in cutaneous wound repair [151]. However, exosomes from HG-pretreated immortalized human epidermal (HaCaT) cells (HG-exos) suppressed angiogenesis through the LINC01435/YY1/HDAC8 axis, thus slowing the rate of wound closure in diabetic mice [67]. Li et al. isolated exosomes from modified placental mesenchymal stem cells (PMSCs) transduced with silk fibroin binding peptide (SFBP)-Gluc-MS2 (SGM) or pac-miR146a-pac protein. The stability and binding rate of SGM-exos were significantly enhanced by the silk fibroin patch (SFP). Engineered exosomes that were altered to adhere to SFP accelerated the healing of diabetic wounds [152]. In addition, Sun et al. presented an adaptable integrated nanoagent based on organic frameworks encased with antibacterial immunoengineered exosomes (PCOF@E-exos). After TNF-α-treated MSCs were exposed to hypoxia, the E-exos were extracted and enclosed in cationic antimicrobial carbon dots (CDs). PCOF@E-exos might work in concert to eliminate bacterial infection, promote angiogenesis, and control oxidative damage as well as inflammation, which hastens wound closure in infected diabetic mice [153] (Table 3).

###### Direct exosome engineering

Several techniques, including electroporation, sonication, freeze–thaw cycles, transfection, extrusion, chimeric exosome technology, and endogenous loading, may be used to load small molecules or medications into exosomes. Various loading techniques affect the loading efficiency of molecules or drugs into exosomes [154]. Fibroblast-derived exosome-mimetic vesicles that can carry matrine (MHEM) were created by gradient extrusion. In the inflammatory milieu, MHEM improved angiogenesis, cell migration, and tissue regeneration during wound healing [155]. MiR-21-5p mimics were electroporated into hADSC-exos as a possible diabetic wound treatment option. Through Wnt/β-catenin signaling, hADSC-exos accelerated diabetic wound healing *in vivo* by inducing vascular neogenesis, reepithelialization, and collagen remodeling [156]. Similarly, exo-miR-146a was created by loading miR-146a-5p into BMSC-exos using electroporation. By encouraging M2 macrophage polarization and HUVEC viability through a reduction in tumor necrosis factor receptor-associated factor 6 (TRAF6) expression, exo-miR-146a enhanced refractory diabetic wound healing [157]. Engineered miR-31 exosomes facilitated diabetic wound healing by enhancing vascular neogenesis, reepithelization and fibrogenesis *in vivo* [158]. Zhao et al. utilized an EXPLOR system to load a significant amount of eNOS into UCMSC-exos when they were exposed to blue light. These engineered UCMSC-exos markedly improved the inflammatory profile and altered the surrounding immune milieu, in addition to greatly increasing the pace of wound closure in diabetic mice [159]. Engineering tactics may increase the loading efficiency of exosomes. For example, the loading effectiveness of miR146a in SGM-miR146a-exos was 10 times greater than that of miR146a-exos alone. SGM-miR146a-Exo@SFP promoted diabetic wound healing with anti-inflammatory and regenerative effects [152].

#### Challenges associated with exosome application

The five most common exosome processing procedures are ultracentrifugation, precipitation, ultrafiltration, immunoaffinity capture, and size-exclusion chromatography. According to a recent meta-analysis of EVs in wound healing, the most popular method was ultracentrifugation (n = 43, 63.2%), which involves a variety of centrifugation regimens. Size exclusion chromatography (SEC), density gradient ultracentrifugation, and commercial precipitation-based isolation kits were utilized in 2 (2.9%), 6 (8.8%), and 16 (23.5%) investigations, respectively. Microfluidics, asymmetrical flow field flow fractionation, and tangential flow filtration (TFF) were not employed in any study. To achieve greater purity, 32 studies (47.1%) utilized two or more separation methods [142]. Exosome preparation methods for diabetic wounds are not highly standardized, and there is significant variation in exosome isolation techniques across studies. In addition, increasing the production of exosomes involves increasing the growth of cells as well as the extraction and purification of exosomes from conditioned media. While there are various approaches for separating exosomes from conditioned media, only two are acceptable for therapeutic use: dUC and SEC [9]. Nevertheless, scaling up exosome manufacturing for clinical use is currently difficult. Quality control (QC) is a critical step in guaranteeing that exosomes can be used repeatedly in a research context and ensuring the purity, uniformity, and integrity of exosome products before they are utilized in therapies. As per the guidelines set forth by the International Society for Extracellular Vesicles (ISEV), the QC of exosomes should be performed for key attributes such as number, size, marker, collection, and purification [160,161]. However, universal standardization of the isolation, optimization, and purification of exosomes is still lacking [162]. Moreover, exosome potency is regarded as a significant criterion for controlling the quality of exosomes in therapeutic applications. However, no gold standard methodologies for potency testing have been proposed yet [9]. The contents of exosomes, which include nucleic acids, lipids, proteins, and metabolic enzymes, largely rely on the surrounding environment and the metabolic conditions of host cells [29]. Nevertheless, very little is known about their mechanisms, functions, and interactions. Further studies are required to determine the exact molecular pathways and the involvement of exosomes in the healing and cutaneous regeneration of diabetic wounds, despite the abundance of studies in these areas. In-depth research is required in the fields of exosome biogenesis, isolation, characterization, cellular uptake, and engineering before the materials can be utilized as diagnostic or therapeutic tools. In addition to transferring molecules to regulate the physiological conditions of target cells, exosomes can be employed to transport therapeutic reagents with high cargo loading because of their nanoscale size [22]. Engineering strategies to obtain modified exosomes may focus on manipulating parent cells before exosome separation, isolating exosomes, packaging therapeutic reagents into exosomes, and delivering exosomes, all of which require additional investigations.

## Conclusions

Exos provide excellent prospects for diabetic wound healing and regeneration. The distinct and innate biological characteristics of these materials allow them to have excellent tissue-homing ability, biocompatibility, circulatory stability, and low immunogenicity. Exos can affect immunological responses and inflammation, accelerate proliferation, stimulate angiogenesis, and regulate collagen remodeling. The ability of exosomes to promote diabetic wound healing is enhanced when they are combined with hydrogels or dressings. Exos are desirable agents for treating diabetic wounds, and we should concentrate on their content that yields the best therapeutic results in the future. However, there is still a poor concordance rate in animal models. While most of the biological properties of exosomes are inherited from their parent cells, their mechanisms of biogenesis and intercellular communication remain largely unknown. Challenges such as the scarcity of clinical-grade exosomes, quality control, and standardization of potency tests impede the clinical utilization of exosomes. Only a few clinical trials have evaluated the therapeutic benefits of exosomes for diabetic wound treatment, and the relevant medications have yet to be licensed for usage. Future research will be required to ascertain the range of applications for exosomes as well as their methods of synthesis for a variety of therapeutic applications. Engineering strategies to modify exosomes and expand their application are also worth exploring.

## Abbreviations

ECM, Extracellular matrix; DFU, Diabetic foot ulcer; DM, Diabetes mellitus; EVs, Extracellular vesicles; mRNAs, Messenger RNAs; miRNAs, MicroRNAs; tRNAs, Transfer RNAs; lncRNAs, Long noncoding RNAs; mtRNAs, Mitochondrial DNA; MVBs, Multivesicular bodies; ILVs, lntraluminal vesicles; DLS, Dynamic light scattering; NTA, Nanoparticle tracking analysis; TEM, Transmission electron microscopy; AFM, Atomic force microscopy; FACS, Flow cytometry; TRPS, Tunable resistive pulse sensing; SPR, Surface plasmon resonance; DLD, Deterministic lateral displacement; ECs, Endothelial cells; MSCs, Mesenchymal stem cells; BMSCs, Bone marrow mesenchymal stem cells; ADSC, Adipose mesenchymal stem cell; ADSC-exos, ADSC-derived exosomes; FGF-2, Fibroblast growth factor 2; PDGF-BB, Platelet-derived growth factor BB; UCMSCs, Umbilical cord mesenchymal stem cells; TGF-β, Transforming growth factor beta; a-SMA, Smooth muscle actin; MMPs, Matrix metalloproteinases; PKC, Protein kinase C.; KLF2, Kruppel-like factor 2; ceRNA, Competing endogenous RNA; ECH, Electroconductive hydrogel; BMSC-exos, BMSC-derived exosomes; ECH-Exos, ECH loaded with BMSCs-exos; DHC-BME, Hyaluronan-collagen hydrogel loaded with BMSC-exos; MSC-exos, Mesenchymal stem cell-derived exosomes; siRel, c-Rel-specific siRNA; MenSC, menstrual blood-derived mesenchymal stem cell; ESC, Epidermal stem cell; ESC-exos, ESC-derived exosomes; PA, *P. americana* L; PAELNs, PA-derived exosome-like nanoparticles; FGF-7, Fibroblast growth factor-7; HSP90, Heat shock protein 90; STZ, Streptozotocin; VEGF, Vascular endothelial growth factor; HG, High glucose; EPCs, Endothelial progenitor cells; ROS, Reactive oxygen species; HUVECs, Human umbilical vein endothelial cells; VH-EVs, ESC-EVs loaded with VH298; NVs, Nanovesicles; HIF-1α, Hypoxia inducible factor-1α; VEGFA, Vascular endothelial growth factor A; USC-exos, Urine-derived stem cells; DMBT1, Deleted in malignant brain tumors 1; HUCMSC, Human umbilical cord mesenchymal stem cell; HUC-exos, HUCMSC-derived exosomes; HDAC8, Histone deacetylase 8; Serum-exos, Serum exosomes; PBS, Phosphate-buffered saline; NC, Negative control; hADSC-exos, Exosomes from human adipose-derived mesenchymal stem cell; MEs, M2 macrophage-derived exosomes; FHE@exos, FHE@exosomes; FEP@exos, FEP@exosomes; ADSC-HExos, Hypoxia-pretreated ADSC-Exos; GelMA, Methacrylate gelatin; GelMA-HExos, ADSC-HExos-embedded GelMA hydrogel; PF-127, Pluronic F-127; PVA, Polyvinyl alcohol; exo@H, HUC-exos encapsulated in PVA/alginate nanohydrogel; MNs, Microneedles; PMN, Polymorphonuclear neutrophil; aPMNEM, PMN exosome mimetics; Gel-VH-EVs, GelMA hydrogel containing VH-EVs; PUAO, Antioxidant polyurethane; hAAM, Human acellular amniotic membrane; HDFs, Human dermal fibroblasts SA, Alginate hydrogel; PDA, Polydopamine; MEs@PMN, MEs encapsulated in needle tips and PDA; vWF, von Willebrand Factor; SP, Silk protein; PRP, Platelet-rich plasma; FG, Fibrin scaffold; 3D, Three-dimensional; 3D-PT-P/GM, Nanofiber/hydrogel core-shell scaffold with 3D multilayer patterned structure; MeHA, Methacrylated hyaluronic acid; eNOS, Nitric oxide synthases; NO, Nitric oxide; SNAP, S-nitroso-N-acetyl-D,L-penicillamine; PNO, NO microbubble-captured hydrogel; PPGA, Poly(PEGMA-co-GMA-co-AAm); HBPL, Hyperbranched poly-L-lysine; MnO2, Manganese dioxide; PCL, Polycaprolactone; GSNO, S-nitrosoglutathione; CMC, Carboxymethyl chitosan; ALg, Sodium alginate; CMC-ALg-GSNO, GSNO loaded into CMC/ALg composite film; CSA, Adenine-modified chitosan; CST, Thymine-modified chitosan; bArg, binary l-arginine; CAT/bArg/GSON Hydrogel that combined CSA and CST with GSNO and bArg; HKUST-1, Copper-benzene-1,3,5-tricarboxylate; MOF, Metal–organic framework; GO, Graphene oxide; NHGs, NO@HKUST-1@GO microparticles; PANI, Polyaniline; HTCC, Hydroxypropyltrimethyl ammonium chloride chitosan; NIR, Near-infrared; PTAT, Photothermal antibacterial therapy; HA, Hyaluronic acid; Gel-HAB, HA hydrogel dressing; AMNP-BNN6, Gel-HAB loaded with allomelanin (AMNP)-N, N′-dis-sec-butyl-N, N′-dinitroso-1, 4-phenylenediamine (BNN6) nanoparticles; GOx, Glucose oxidase; L-Arg, L-arginine; H2O2, Hydrogen peroxide; HA-DA, Hyaluronic acid-graft-dopamine; HbO2/H2O2, Oxyhemoglobin/hydrogen; CvMN, Cv-loaded microneedles; CS-PVA, Chitosan-polyvinyl alcohol; CPO, Calcium peroxide; CuP, Copper hydrogen phosphate; pEVs, Platelet EVs; LEAP, Ligand-based Exosome Affinity Purification; CCUs, Chronic skin ulcers; WJ-MSCs, Wharton’s jelly mesenchymal stem cells; PEP, Purified exosome product; CLECs, Human umbilical cord lining epithelial cells; HSGs, Human skin allografts; KLF4, KLF transcription factor 4; MT-exos, Melatonin-pretreated MSC-exos; PGZ-exos, Pioglitazone-pretreated MSC-exos; ATV-exos, Atorvastatin-pretreated MSC-exos; IFN-γ, Interferon-γ; LPS pre-exos, LPS-preconditioned MSC-exos; HaCaT cells, HG-pretreated immortalized human epidermal cells; HG-exos, Exos from HaCaT cells; PMSCs, Placental mesenchymal stem cells; SFBP, Silk fibroin binding peptide; SGM, SFBP-Gluc-MS2; SFP, Silk fibroin patch; PCOF@E-exos, Organic nanoagents encased with antibacterial immunoengineered exos; CDs, Carbon dots; MHEM, Matrine; TRAF6, Tumor necrosis factor receptor-associated factor 6; SEC, size-exclusion chromatography; TFF, Tangential flow filtration; QC, Quality control; ISEV, International Society for Extracellular Vesicles.

## Ethics approval and consent to participate

The authors declare that their participation in writing this review and its publication are voluntary.

## Consent for publication

Not applicable.

## Data Availability

Not applicable.
